# Time course for urethral neuromuscular reestablishment and its facilitated recovery by transcutaneous neuromodulation after simulated birth trauma in rats

**DOI:** 10.1038/s41598-021-01200-x

**Published:** 2021-11-03

**Authors:** José L. Palacios, Ricardo Juárez, Nancy Mirto-Aguilar, Alvaro Munoz, Margot S. Damaser, Yolanda Cruz

**Affiliations:** 1 Doctorado en Ciencias Biológicas, Universidad Autónoma de Tlaxcala, Tlaxcala, Mexico; 2 Doctorado en Ciencias Biomédicas, Universidad de Guadalajara, Guadalajara, Mexico; 3 Doctorado en Investigaciones Cerebrales, Universidad Veracruzana, Veracruzana, Mexico; 4 Instituto de Ciencias, Benemérita Universidad Autónoma de Puebla, Puebla, Mexico; 5 Centro Universitario del Norte, Universidad de Guadalajara, Guadalajara, Mexico; 6 Department of Biomedical Engineering, Lerner Research Institute, Cleveland Clinic, Cleveland, OH USA; 7 Advanced Platform Technology Center, Department of Veterans Affairs Medical Center, Louis Stokes Cleveland, Cleveland, OH USA; 8 Centro Tlaxcala de Biología de La Conducta, Universidad Autónoma de Tlaxcala, Carretera Federal Tlaxcala-Puebla Km 1.5, Zip Code 90070, Tlaxcala, Mexico

**Keywords:** Systems biology, Anatomy, Neurology, Urology

## Abstract

The aims of the study were to determine the time-course of urinary incontinence recovery after vaginal distension (VD), elucidate the mechanisms of injury from VD leading to external urethral sphincter (EUS) dysfunction, and assess if transcutaneous electrical stimulation (TENS) of the dorsal nerve of the clitoris facilitates recovery of urinary continence after VD. Rats underwent 4-h VD, 4-h sham VD (SH-VD), VD plus 1-h DNC TENS, and VD plus 1-h sham TENS (SH-TENS). TENS or SH-TENS were applied immediately and at days 2 and 4 post-VD. Micturition behavior, urethral histochemistry and histology, EUS and nerve electrophysiology, and cystometrograms were evaluated. VD induced urine leakage and significantly disrupted EUS fibers and nerve-conduction (VD vs SH-VD group*;* *p* < 0.01). Urine leakage disappeared 13 days post-VD (*p* < 0.001). Structural and functional recovery of EUS neuromuscular circuitry started by day 6 post-VD, but did not fully recover by day 11 post-VD (*p* > 0.05). TENS significantly decreased the frequency of urine leakage post-VD (days 5–7*;* *p* < 0.01). We conclude that rat urinary continence after VD requires 2 weeks to recover, although urethra structure is not fully recovered. TENS facilitated urinary continence recovery after VD. Additional studies are necessary to assess if TENS could be used in postpartum women.

## Introduction

Prolonged childbirth is a risk factor for urogenital disorders, such as urinary incontinence and sexual dysfunction that affect health and quality of life^1,2^. In rats, a vaginal distension (VD) model has been created to better understand the injury processes during childbirth in women^3,4^. VD induces bladder and vaginal hypoxia, damage to urethral musculature, decrease in urethral pressure, temporary abolishment of electromyographic (EMG) activity of the external urethral sphincter (EUS)^5^, as well as behavioral signs of stress urinary incontinence within 3 days^6^. Considering that rats are the most used laboratory animal for urological studies, greater knowledge of the mechanisms of urethral somatic dysfunction and time course of urinary incontinence recovery after VD may help to assess novel treatments to accelerate recovery of urinary continence.

Peripheral nerve electrical stimulation (1 h, 20 Hz), proximal to the nerve injury, enhances nerve regeneration^7^. Similarly, in a combinatory injury of VD and pudendal nerve (PDN) crush in rats, 1 h of electrical stimulation of the PDN (20 Hz, 0.3 mA, 0.1-ms pulse), proximal to the crushed region, increased expression of BDNF and βII-tubulin in EUS motoneurons^8,9^, suggesting that electrical nerve stimulation induces neuro-regenerative processes of the EUS neural circuitry after childbirth-like trauma. Multiple treatments of electrical stimulation appear to further accelerate recovery in this same model^10^. However, electrical stimulation of the PDN is invasive. Therefore, a non-invasive electrical nerve stimulation is desirable for clinical translation of this treatment.

Given that the EUS can also be reflexively activated by stimulation of perineal skin^11^, transcutaneous electrical nerve stimulation (TENS) of the dorsal nerve of the clitoris (DNC) of VD animals^12^ will synaptically recruit motoneurons of Onuf’s nucleus and induce a neuro-regenerative process in PDN motor axons. Thus, we hypothesize that TENS facilitates urinary continence recovery after childbirth damage.

The aims of the present study were to determine in female rats, the time course of behavioral urinary continence recovery after VD, elucidate the mechanisms of EUS dysfunction after VD and assess if TENS of the DNC facilitates recovery of urinary continence subsequent to VD. Some results of this study were presented in abstract form^13^.

## Materials and methods

### Animals

Experiments were conducted using ninety six adult nulliparous Wistar female rats (250–300 g, CTBC vivarium) in accordance with the guidelines of the Mexican Council on Laboratory Animal Care (NOM-062-ZOO-1999), Guide for the Care and Use of Laboratory Animals (National Research Council 2011, USA) and institutional Ethical approval (Autonomous University of Tlaxcala Laboratory Animal Welfare and Ethical Review Committee) in compliance with the ARRIVE guidelines.

### Experimental design

Animals were housed with water and food ad libitum and maintained on a 12:12-h light–dark cycle. Rats were randomized to undergo VD, sham VD (SH-VD), VD with TENS of the DNC (TENS) or VD with sham TENS (SH-TENS). The VD and SH-VD procedures were performed as previously reported^6^. Briefly, in the VD group, animals were anesthetized with a mixture of intraperitoneal ketamine (60 mg/kg; Cheminova, Mexico) and xylazine (7.5 mg/kg; Pisa, Mexico), and additional doses were used as needed. For catheter implant, a cotton swab moistened with mineral oil was introduced into the vagina. A modified 10-F latex sterile Foley balloon catheter was then inserted into the vagina, inflated with 4 ml water, and left in place for 4 h. In SH animals, the catheter was placed into the vagina but was not inflated. The catheter was secured with a double silk suture at the skin of the perivaginal orifice. After VD or SH-VD, experimental parameters were evaluated at different time-points as described next.

Micturition behavior of the rats was video-recorded and analyzed for 6 h in the dark phase as described in the description of Experiment 1. The urethra and vagina of VD or SH-VD animals were collected and stained with Masson's Trichrome or Acetylcholinesterase (AchE) histochemistry, as described in detail in the description of Experiment 2. Electroneurography (ENG) of the afferent and efferent nerves of the pudendal nerve, EUS EMG and cystometrograms (CMGs) were evaluated in urethane anesthetized rats (1.2 g/kg, i. p.) using electrophysiological and urodynamic systems, as detailed in the description of Experiment 3. For TENS, three sessions of electrical stimulation of the clitoral sheath using a pair of electrodes were performed immediately and at 2 and 4 days post-VD. In SH-TENS animals, electrical stimulation was not delivered. Details of the stimulation parameters are given in the description of Experiment 4. In both groups, micturition behavior of the rats was video-recorded and analyzed for 6 h corresponding to the dark phase, starting from day 5 post-VD.

### Experiment 1. Time course for urinary continence behavior recovery

In order to determine the time period required to recover urinary continence after VD, micturition behavior of SH-VD (n = 5) and VD (n = 8) rats was recorded before and every two days after VD, until leakage was no longer observed (3 to 13 days after VD). Rats were videotaped using an infrared camera connected to a digital video recording system (Hikvision, China) during the last 6 h of the dark phase and micturition behavior was recorded as previously described^6^. Briefly, rats were located in an evaluation cage consisting of a standard transparent housing cage with water and food in the middle of the cage and the plexiglas floor replaced with a wire grid supported 4 cm above a glass-topped stand. A urine collector plate was placed in the 4-cm space and could be slid out from below the cage to collect and measure the expelled urine using an insulin syringe. A void was considered as valid when urine expulsion (volume > 0.20 ml) was associated with micturition behavior^6^. Drops were considered as any urine expelled (volume ~ 0.02 ml) without stereotyped micturition behavior as defined in a prior publication^6^. The number of drops in 6 h, frequency of voids in 6 h, void duration (time for urine expulsion), voiding interval and voided volume were determined.

### Experiment 2. Time course for urethral structure recovery

Histological and histochemistry techniques were used to determine the time course of VD-induced urethral damage and recovery. The studies were performed immediately after VD, and on days 3, 5, 11, and 12 after VD or SH-VD. For histology, rats (n = 12, three animals per group) were anesthetized (urethane 1.2 g/Kg, ip; Sigma-Aldrich St. Louis, MO) and transcardially perfused with saline followed by 10% formalin and the pelvic urogenital tract collected as described previously^14^. The pelvic region of the urethra was dissected, dehydrated in alcohol (70–100%) and xylol (100%), embedded in paraplast X-tra, and sectioned with a microtome (Leica, RM2125RT) at 7 μm thickness (transversal sections). Sections were stained with Masson's Trichrome^14^ and examined with an optical microscope (Axio Imager A1, Carl Zeiss, Thornwood, NY). Representative sections were photographed with a digital camera (Cannon PowerShot S50, Canon USA, Lake Success, NY), at low (5X and 20X) and high magnification (100X). The morphometric characteristics of the pelvic urethra, including structure and signs of injured tissue were analyzed. The thickness of the epithelium layer and the fragmentations of the striated fibers were analyzed in one section of the dorsal region of the urethra per animal. Measurements were made using AxioVision image processing software (version 4.6, Carl Zeiss). Epithelium layer was manually outlined at 100X magnification for accurate delimitation. Fragmentations were considered as discontinuities or ruptures between striated circular fibers, and their number was estimated on an area corresponding to 20,000 µm^2^.

AchE histochemistry (n = 12; three rats per group) was used to mark cholinergic innervation of the EUS. It was performed as reported elsewhere^14^. Briefly, urethane-anesthetized rats were transcardially perfused with 300 mL of saline solution (0.9% NaCl). The urogenital tracts were dissected and a longitudinal incision was made in the dorsal vaginal-wall to extend it flat, and fat tissue removed (all samples were processed simultaneously). Thereafter, the urogenital tracts were fixed by immersion in 10% formalin for at least 48 h. Then, the urogenital tracts tissues were washed in PBS, incubated for 2.5–3 h at room temperature with a solution consisting of acetylthiocholine-iodide dissolved in 0.1 M sodium-hydrogen-maleate, 0.1 M sodium-citrate, 30 mM CuSO4, 5 mM potassium-ferricyanide, and 0.1% Triton X-100 (pH 6.0). Tissues were rinsed in distilled water, dehydrated with ascending alcohol concentrations, cleared with xylene, and the urethras were observed under a stereoscopic microscope (Leica M80) and photographed with a digital camera (Cannon PowerShot S50, Canon USA, Lake Success, NY). Measurements of the AchE positive area in the urethra were analyzed using AxioVision image processing software (version 4.6, Carl Zeiss).

### Experiment 3. Time course for the EUS neuromuscular circuitry recovery

The integrity of the EUS neuromuscular circuitry was determined in SH-VD and VD urethane anesthetized rats (1.2 g/Kg, i.p.) using electrophysiological techniques. Immediately (n = 5) and three days (n = 5) after VD, or sham-VD (n = 5), the functionality of the afferent and efferent components of the EUS-clitoral reflex was determined by ENGs recordings of the DNC (at the level of the ischiatic arch) and the motor branch of the pudendal nerve (MBPD). The latter was recorded at two places: at the small branches localized close to the urethra after MBPD bifurcation (EUS branch, EUSb), and at the middle region of the ischiorectal fossa. Simultaneously to the ENGs, EMGs of the EUS were recorded. The ENGs and EMGs were recorded before and during gentle squeezing of the clitoral sheath with Adson forceps without teeth (~ 10–15 g-force for ~ 2 s) to trigger the clitoral-EUS reflex.

**Figure 1:**
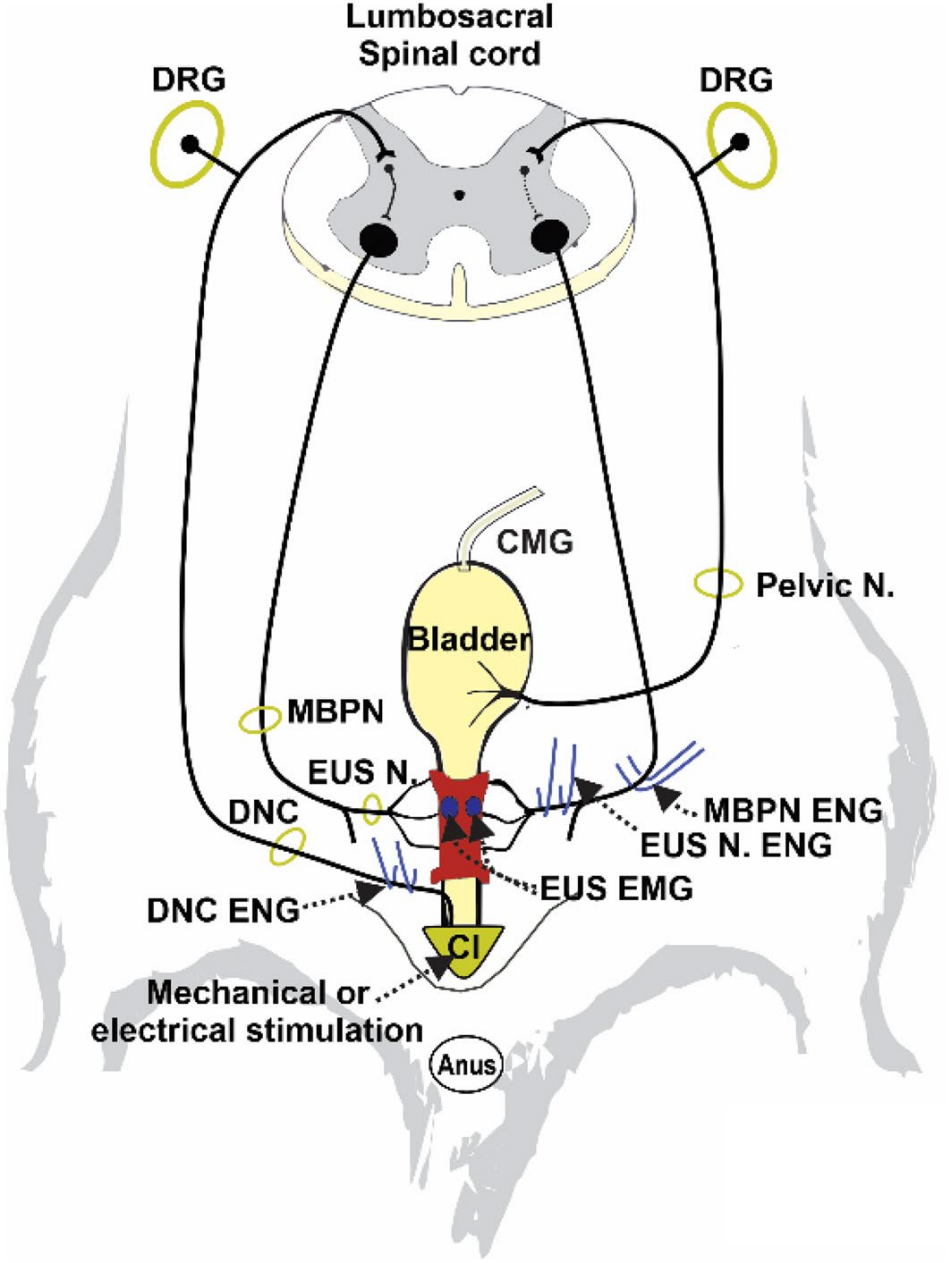
Diagram showing a ventral view of the lower urinary tract of the female rat and its peripheral and central neural control. Blue lines and small circles indicate electrode placement for electroneurograms (ENG) and EUS electromyograms (EMG), respectively, during clitoral mechanical stimulation and cystometrograms (CMG). DRG, dorsal root ganglia; MBPN, motor branch of the pudendal nerve; EUS N, external urethral sphincter nerve; DNC, dorsal nerve of the clitoris; Cl, clitoris.

ENGs were recorded as described elsewhere^6^. Briefly, nerves were dissected at the above described different anatomical levels (Fig. 1) and mounted on platinum bipolar hook-electrodes, which were connected to a Grass amplifier (P51, USA, bandpass filter between 100 Hz to 3 kHz). The amplifier was connected to an electrophysiological recording system (Digidata 1440A, 10 kHz sampling rate, Molecular Devices, USA).

For recording EUS EMG signals the pubic bone was removed and, using a stereomicroscope (Leica M80, Germany), the tips of a pair of teflon-coated stainless steel wires (uncoated diameter 0.005″, bared for ~ 1 mm at the tip, A-M Systems, USA) were inserted into the EUS. The electrodes were connected to a Grass amplifier (P51, USA, bandpass filtered between 100 Hz and 3 kHz) which was connected to an electrophysiological recording system (Digidata 1440A, 10 kHz sampling rate, Molecular Devices, USA). These devices allowed for compound nerve potentials to be stored and analyzed.

Recovery of functionality of the EUS neuromuscular circuitry after VD was evaluated in urethane anesthetized rats (1.2 g/Kg, i.p.) by recording EUS EMG during the clitoral-EUS reflex and during cystometry (n = 32); immediately and on days 3, 6, 7, 8, 9, 10, and 11 after VD or SH-VD. The clitoral reflex was evoked by gently squeezing the clitoral sheath as mentioned above. The clitoral reflex was induced at least 5 times per animal. Then, after a period of 15 min the animals were prepared for cystometry. Cystometrograms (CMGs) and EMGs were simultaneously recorded as previously reported^15^. Briefly, a silastic cannula (1.5 mm outer diameter) was inserted into the bladder dome and secured with a purse-string suture. This suprapubic catheter was connected to pressure transducer and a syringe pump to infuse saline (5 ml/h). Continuous CMGs were recorded for 30 min. The field potentials recorded before clitoral stimulation or before a bladder contraction were considered as the basal EUS EMG activity.

ENGs and EMGs were analyzed using Clampfit 10.4 software (Molecular Devices, USA).

The amplitude and frequency of firing in 1-s samples identified at the middle of the EUS evoked response were determined. The average of three samples per animal was used for statistical analysis. Since the amplitude of the EUS-EMG of the SH-VD rats was not significantly different between the different assessed days (*p* > 0.05), average EUS EMG amplitude of SH-VD rats at all time points was considered to be 100% and compared to the values recorded from VD rats.

In each rat, the last three bladder contractions from continuous CMGs were analyzed to determine voiding efficiency, intercontractile interval, and amplitude and duration of bladder contraction. Data from the three CMGs in each animal were averaged for statistical analysis. Voiding efficiency was calculated as: voided volume/(voided volume + residual urine) X 100%.

### Experiment 4. Facilitated recovery of urinary continence by TENS of the DNC

Micturition behavior of VD rats was recorded after treatment with TENS (n = 6) or SH-TENS (n = 6). TENS was applied immediately after VD and on days 2 and 4 after VD in anesthetized rats with ketamine (60 mg/kg; Cheminova, Mexico) and xylazine (7.5 mg/kg; Pisa, Mexico). Micturition behavior was recorded from day 5 after VD and continued until urine leakage disappeared. The behavior of the rats was videotaped as described in Experiment 1.

For TENS, the animals were in a supine position and an electroconductive gel (Ultrasonic, Mexico) was applied to the clitoral sheath, which was previously shaved and cleaned. Then, using a micro-manipulator (Narishige, Japan), bipolar stainless steel hook-electrodes were placed on the ventral wall of the clitoral sheath surface (near of the entrance of the external urethral meatus) of the VD rats. The electrodes were connected to an isolation unit (Grass SIU-5, USA), which in turn was connected to a stimulator (Grass S48, USA). Thereafter, electrical current was applied for one hour (additional doses of ketamine and xylazine were used as needed) as previously reported^16^, consisting of square pulses with 0.88 mA of current, 20 Hz of frequency and 1-ms of pulse duration^16^. For SH-TENS rats, the electrodes were placed on the clitoral sheath surface for one hour but electrical current was not applied.

### Statistical analysis

Results are presented as mean ± standard error of the mean (SEM). Two Way ANOVA for repeated measures with a Holm-Sidak post-hoc test were used to compare the urinary parameters and the frequency of drops in experiments 1 and 4. One Way ANOVA with Tukey post-hoc tests were employed to compare the AchE positive area in experiment 2, as well as amplitude of DNC firing and EUS EMG, and cystometric parameters among VD and SH-VD in experiment 3. For all comparisons, a value of *p* < 0.05 was considered to indicate a statistically significant difference between groups.

## Results

### Experiment 1. Time course for urinary continence behavior recovery

**Figure 2:**
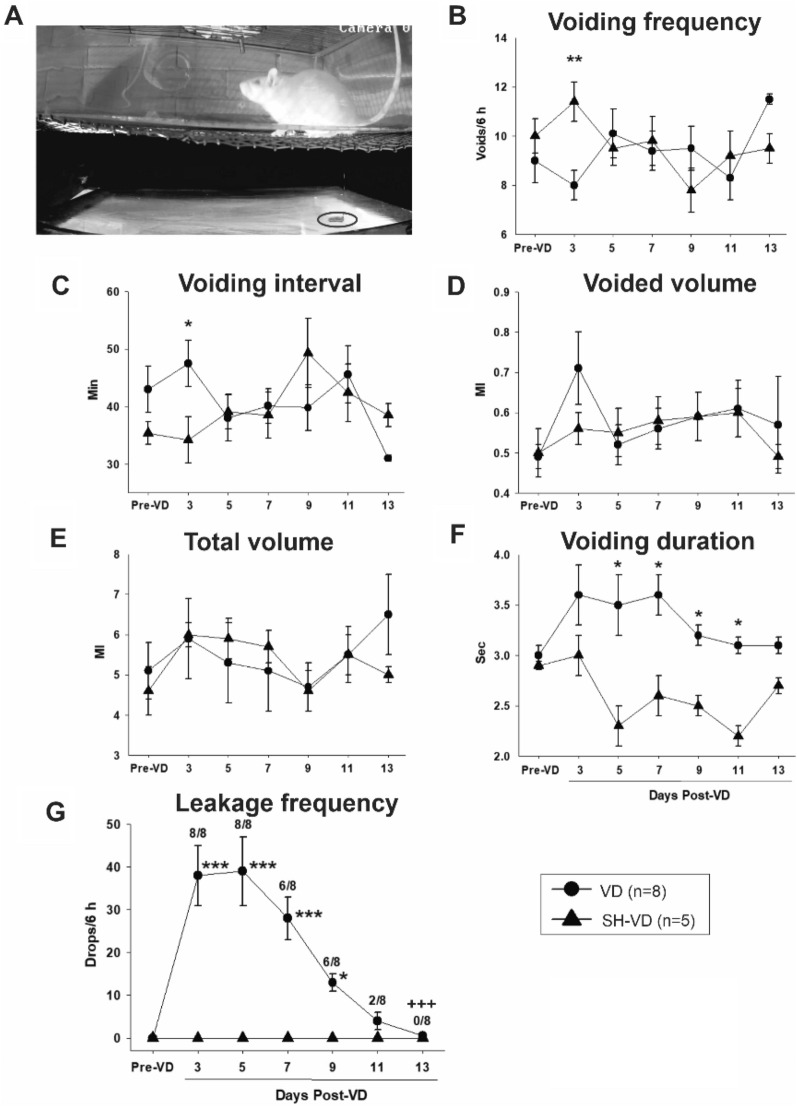
Micturition behavior and urinary parameters of female rats after vaginal distension (VD). (**A**) Photograph depicting stereotyped micturition behavior in the female rat (oval shape indicates urine expelled in the cage corner). Urinary parameters during the dark phase after VD or sham VD (SH-VD) including voiding frequency (**B**), voiding interval (**C**), voided volume (**D**), total voided volume (**E**), and voiding duration (**F**). (**G**) Number of drops in the dark phase after VD or SH-VD at the same time point. Values represent mean ± standard error of the mean. * (*p* < 0.05) and ** (*p* < 0.01) indicate significant differences for VD values vs. SH-VD. +++ (*p* < 0.001) indicates significant differences between VD at day 13 vs VD at days 3–7. Two-way repeated measures ANOVA.

Before VD or SH-VD, all rats voided urine with a stereotyped micturition behavior in the corner of the cage (Fig. 2A). The voiding frequency was 10 ± 0.7 voids/6 h in the VD group and 9 ± 0.9 voids/6 h in the SH-VD group. VD decreased voiding frequency and increased voiding interval 3 days after VD (Fig. 2B,C; *p* < 0.01), but did not significantly affect voided volume or total volume (Fig. 2D,E; *p* > 0.05), and increased voiding duration on days 5, 7, 9 and 11 after VD (Fig. 2F; *p* < 0.05). There were no other significant differences in any voiding parameter between evaluated days for either VD or SH-VD rats (Fig. 2B–F; *p* > 0.05).

Prior to VD or SH-VD, rats did not drip urine in any body-posture at any time-point. However, after VD, 100% of the animals dripped urine in the absence of the stereotyped voiding behavior 3 to 6 days after VD. Following those days, the number of animals dripping urine and the number of drops decreased gradually, and leakage disappeared by day 13 after VD (Fig. 2G; *p* < 0.001). Dripping occurred mainly during behaviors implicating stress incontinence, such as sneezing and standing on hind legs for vertical exploration.

### Experiment 2. Time course for urethral structure recovery

**Figure 3:**
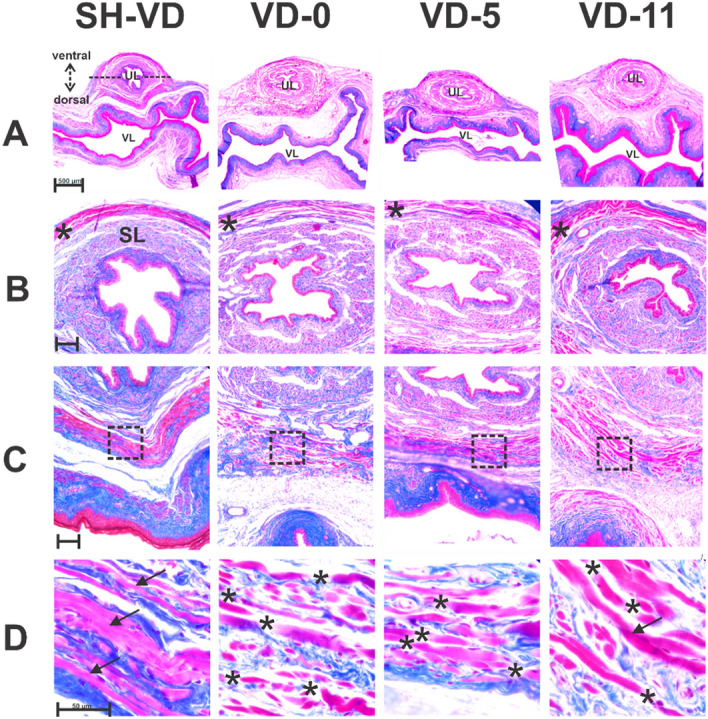
Photos of transverse sections of the urethra of female rats showing time course of structural injuries and recovery after vaginal distension (VD). Sections are stained with Masson´s trichrome and amplified at 5x (**A**), 20x (**B**, ventral side; **C**, dorsal side) and 100x (**D**) magnification. SH-VD, Sham VD; VD-0, immediately after VD; VD-5, at day 5 after VD; VD-11, 11 days after VD. In (**B**), asterisks indicate transverse fibers on the ventral side of the EUS. Photomicrographs in panels D correspond to magnifications of the squares indicated in (**C**), showing the dorsal side of the EUS. Black arrows in (**D**) indicate continuous striated muscle fibers of the EUS, while asterisks in (**D**) indicate discontinuities in EUS muscle fibers. VL, vaginal lumen; SL, smooth muscle; UL, urethral lumen. Scale bars: (**A**) 500 µm; (**B**) 100 µm; (**C**) 100 µm; (**D**) 50 µm.

In SH-VD rats, the EUS and the other urethral layers were compact and well defined. Muscular and epithelial urethral tissue were red (Fig. 3; SH-VD, A-D). Immediately after VD, urethral epithelial thickness was reduced (SH VD = 34 µm ± 2 vs. VD-0 = 22 ± 1.9 µm; *p* < 0.05).

The submucosa and the muscular layers (smooth and striated) were fragmented (Fig. 3A,B). EUS fibers of the urethra were disrupted (no. of disruptions in the dorsal region, SH = 2 ± 0.5; VD-0 = 28 ± 3.7; Fig. 3A–D; *p* < 0.01).

On days 3–5 after VD, fragmentation of the smooth muscle, disruption of striated muscular layers (Fig. 3; VD-5, EUS fibers disruption = 19 ± 2; *p* < 0.05 compared to SH-VD) and thinning of epithelium were still observed (Fig. 3; VD-5, A-D, epithelium layer = 16 µm ± 2.5; *p* < 0.01 vs SH-VD).

By days 11–12 post-VD, the thickness of the urethral epithelia (28 ± 1.8 µm) did not significantly differed from the values obtained in sham animals (*p* > 0.05). However, the integrity of urethral smooth and striated muscle fibers was not fully recovered and signs of fragmentation were still observed (no. of EUS fibers disruption = 12 ± 3; Fig. 3; VD-11, A–D).

**Figure 4:**
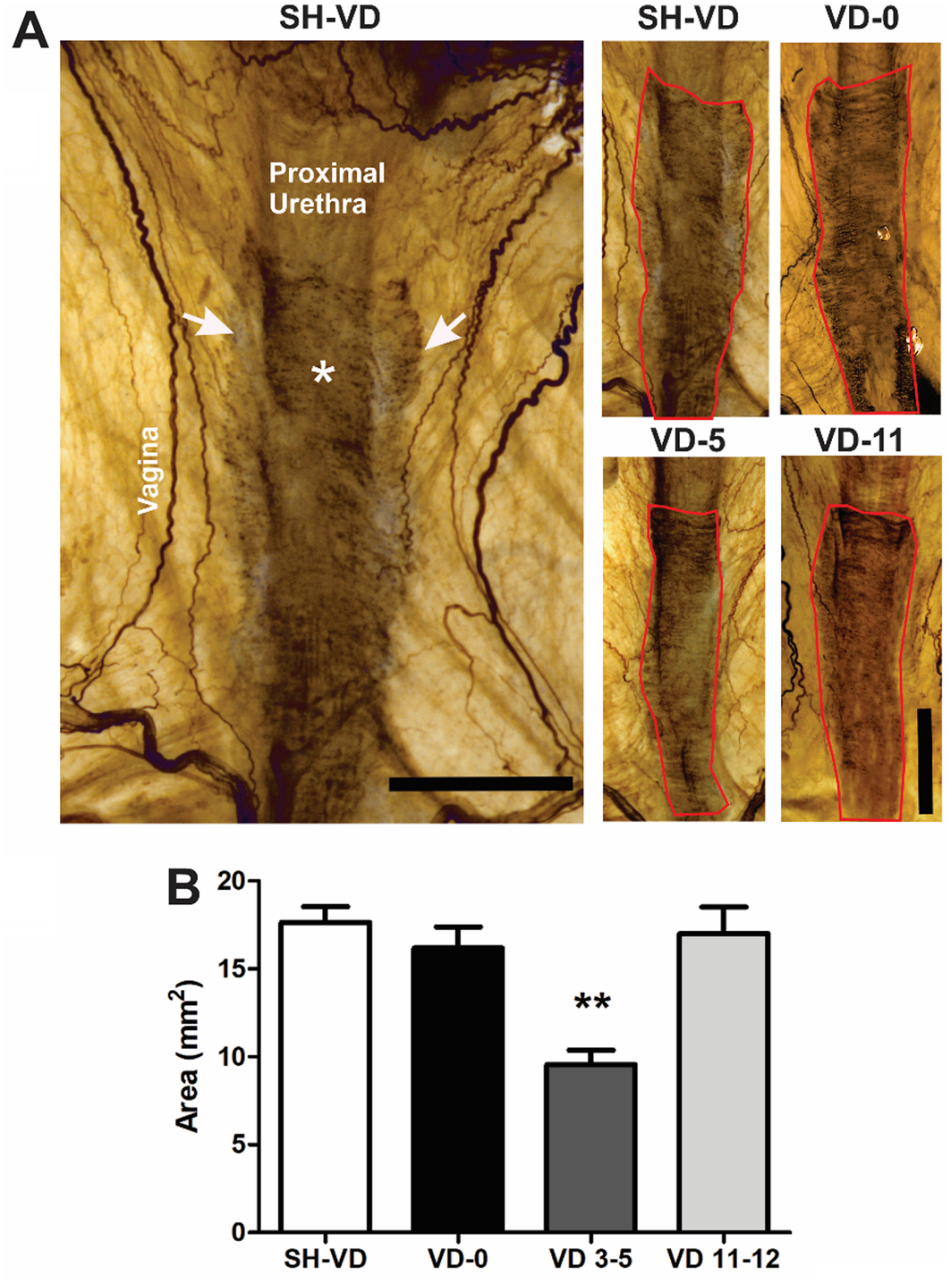
Urogenital tract of rats showing injury and recovery of cholinergic innervation in the urethra after vaginal distension (VD). (**A**) Photos of the urogenital tissue treated with acetylcholinesterase (AchE) histochemistry in a ventral view of urethra from a SH-VD rat, immediately after VD (VD-0), at day 5 (VD-5) or day 11 after VD (VD-11). Red lines illustrate the external urethral sphincter. Asterisk and arrows indicate EUS fibers, both circular and those that attach to the ventral wall of the vagina, respectively. (**B**) Graph of AchE positive areas from SH-VD and VD rats. Values are mean ± standard error of the mean. ** (*p* < 0.01) indicates significant differences among VD at day 5 vs SH-VD, immediate VD, and at day 12. One Way ANOVA. Horizontal and vertical scale bars = 2.5 mm.

In urogenital tracts treated with AchE, nerves were marked as dark brown running on the vaginal wall and in the EUS, the somatic component of the urethra (Fig. 4A). In SH-VD animals, the brown stain indicative of AchE positive nerves and striated muscle fibers depicted a belt shape in the pelvic urethra, with muscle fibers covering the ventral wall of the urethra and its lateral edges attached to the ventral wall of the vagina (Fig. 4A). The AchE area of the EUS was not significantly affected immediately after VD (SH VD = 18 ± 0.8 mm^2^ vs. VD 0 = 16.1 ± 1.1 mm^2^; Fig. 4A,B) but was significantly reduced 3–5 days after VD (9.5 ± 0.8 mm^2^; Fig. 4A,B; *p* < 0.01), especially because the EUS fibers attached to the vagina disappeared. At days 11–12, the area of the AchE was restored to the levels of sham animals (16.3 ± 1.5 mm^2^; Fig. 4A,B).

### Experiment 3. Time course of EUS neuromuscular circuitry recovery

#### Afferent component of the clitoral-EUS reflex

**Figure 5:**
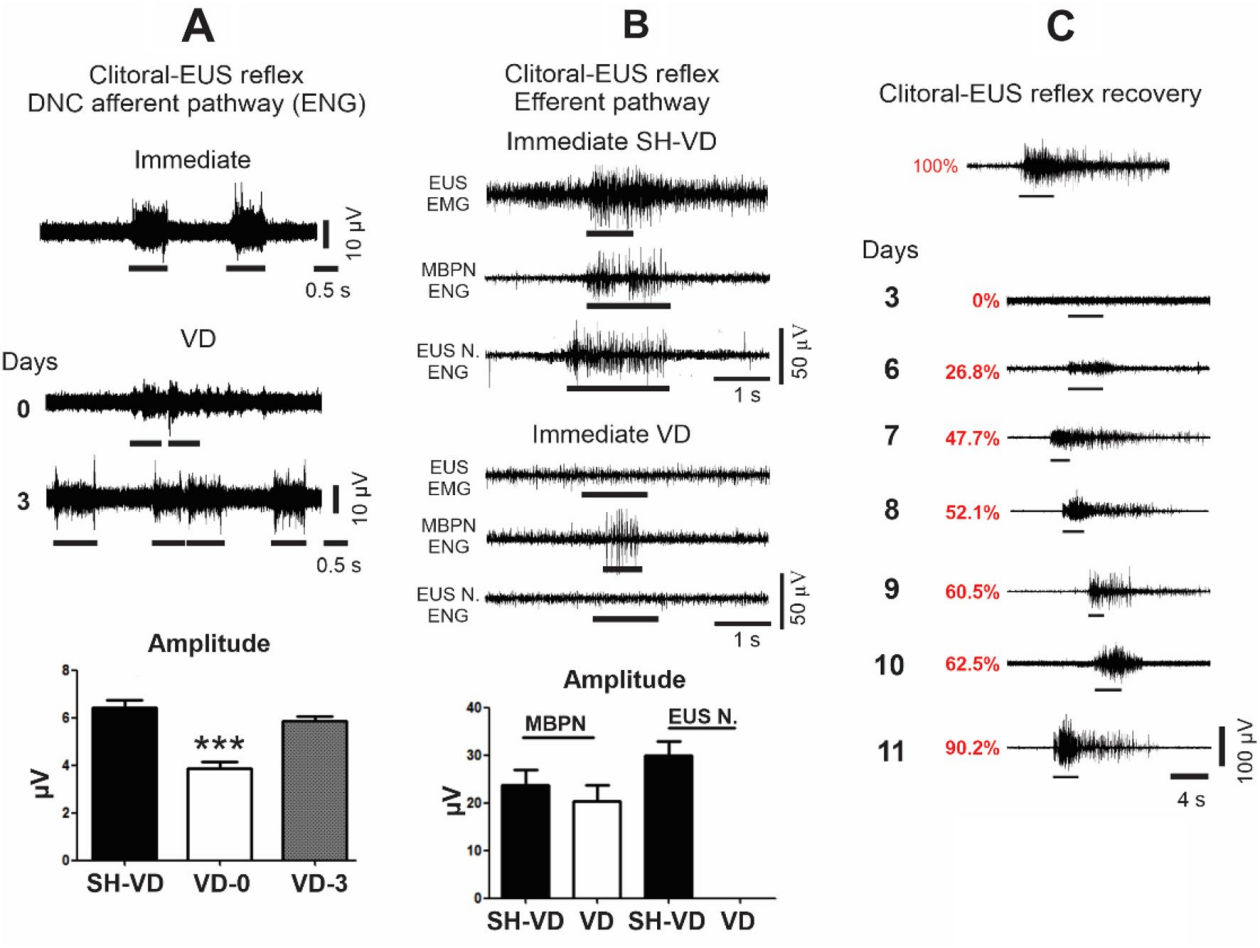
Time course of recovery of the clitoral—external urethral sphincter (EUS) reflex components after vaginal distension (VD) in rats. (**A**) Representative electroneurograms (ENGs) of dorsal nerve of the clitoris (DNC, afferent pathway) immediately and 3 days after VD or sham VD (SH-VD), and graph of the amplitude of DNC activity in SH-VD, immediately, and 3 days after VD. Values are mean ± standard error of the mean. *** (*p* < 0.001) indicates significant differences immediately after VD (VD-0) and SH-VD, and 3 days after VD (One Way ANOVA). (**B**) Representative electromyograms of the external urethral sphincter (EUS EMG), and electroneurograms of the motor branch of the pudendal nerve (MBPN ENG) and the EUS nerve (EUS N. ENG) recorded during mechanical stimulation of the clitoris, immediately after SH-VD or VD. The lower panel shows a graph of the amplitude of MBPN and EUS N. activity in SH-VD, immediately after SH-VD or VD. Values represent mean ± standard error of the mean. (**C**) Representative electromyograms of the external urethral sphincter in SH-VD animals, and at 3, 6, 7, 8, 9, 10 and 11 days after VD.

Immediately after VD (VD0), the amplitude of DNC ENG responses to clitoris mechanical stimulation was significantly decreased compared to that of SH-VD rats (Fig. 5A , *p* < 0.01). However, it recovered to ~ 90% by 3 days after VD (Fig. 5A).

#### Efferent component of the clitoral-EUS reflex

In SH-VD rats, mechanical stimulation of the clitoris (line below ENG or EMG) triggered ENG action potentials in both MBSP and EUS branches, simultaneous with EUS EMG activity (EMG) (Fig. 5B). In contrast, immediately and 3 days after VD, mechanical stimulation of the clitoris did not trigger EUS EMG activity or EUS nerve activity (Fig. 5B). However, the MBPN still responding to the stimulation (MBPN ENG in Fig. 5B). The ENG amplitude of the MBSP of VD rats did not significantly differed of that of SH-VD animals (Fig. 5B).

#### Recovery of the clitoral EUS reflex after VD

In SH-VD rats, the mean amplitude of the EUS EMG activity during clitoral-EUS reflex was 54.2 ± 6 µV (Fig. 5C). Immediately and 3 days after VD, EMG amplitude of the EUS was abolished. Considering average SH-VD value (54.2 ± 6 µV) as 100%, EMG activity decreased to 26.7% (14.5 ± 5 µV) in VD rats by day 6 (Fig. 5C). The amplitude of the EUS EMG in VD rats gradually recovered from 26.7% at day 6 to 90% at day 11 post-VD (Fig. 5C).

#### Bladder EUS reflex during CMG after VD

**Figure 6:**
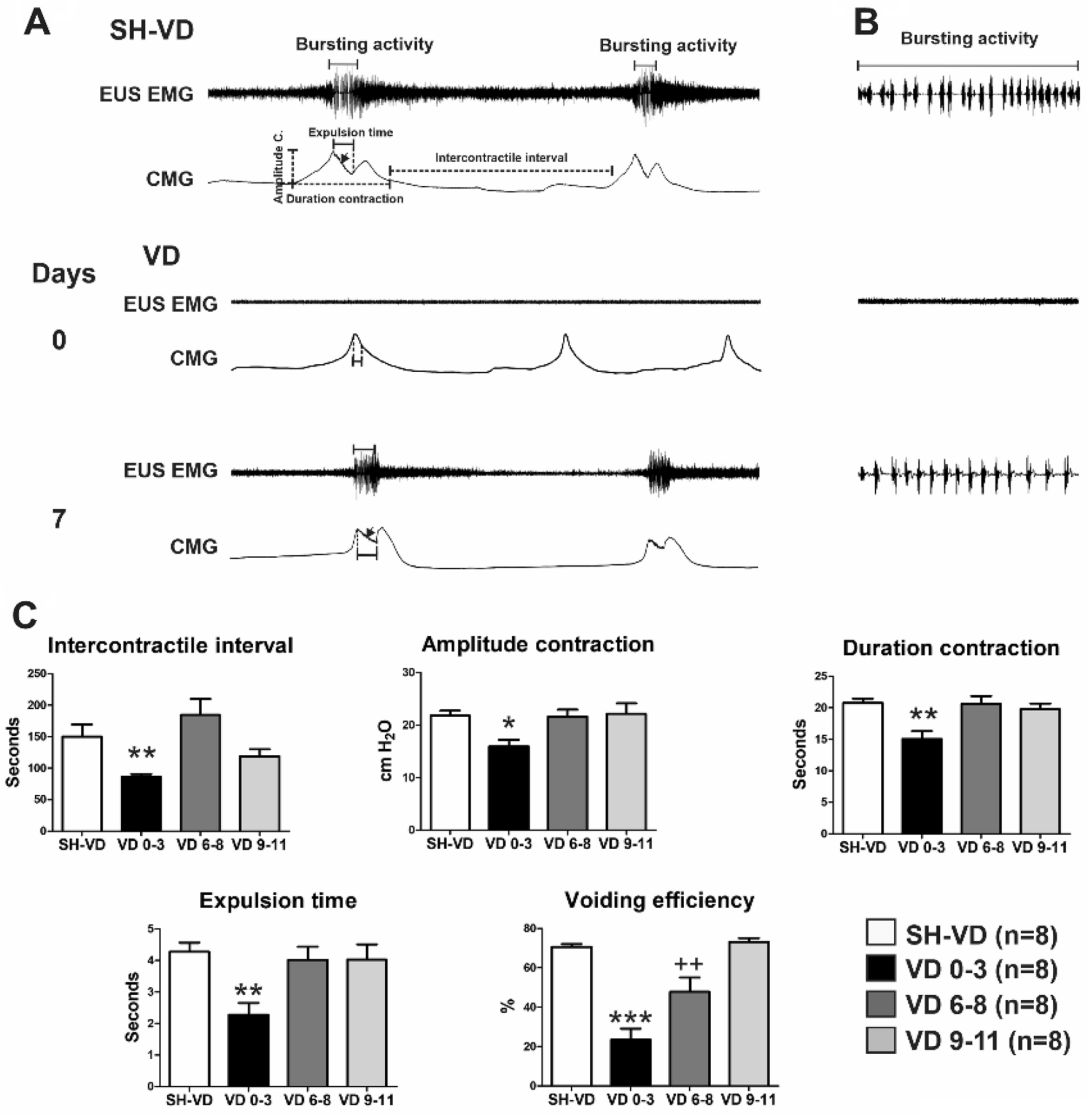
Cystometry and urinary parameters showing time-course of recovery of external urethral sphincter (EUS) and bladder activity after vaginal distension (VD) and sham VD (SH-VD). (**A**) Representative electromyograms (EMGs) with tonic and bursting activity of the EUS, and bladder contractions at voiding during cystometry in SH-VD rats (upper trace), and following 0 and 7 days after VD. The black arrow indicates the presence of intraluminal pressure high-frequency oscillations. (**B**) Representative EMGs of the EUS with phasic pattern activity during urine expulsion at voiding while performing simultaneous cystometry in SH-VD rats (upper trace in **A**), and immediately after VD, and at day 7 following VD. (**C**) Graphs depicting cystometric parameters from SH-VD and VD rats from immediate to 3, 6–8 and 9–11 days post-VD. Values are mean ± standard error of the mean. * (*p* < 0.05), ** (*p* < 0.01) and *** (*p* < 0.001) indicate significant differences for 0–3 days after VD vs. SH-VD and VD on days 6–8 and 9–11. ++ (*p* < 0.01) indicates significant differences at 6–8 days after VD vs. SH-VD rats (One Way ANOVA).

Immediately after SH-VD the EUS discharged tonic and bursting EMG activity during bladder contraction, the amplitude was 93 ± 7 µV and 108 ± 9 µV, respectively (Fig. 6A,B). Immediately after VD (day 0, Fig. 6A,B) and during the first 3 days after VD, the EUS EMG activity was abolished. By day 7 post-VD the amplitude of tonic and bursting EUS EMG activity during bladder contraction was partially recovered; 26 ± 3 µV and 30 ± 5 µV, respectively (Fig. 6A,B). The EUS response gradually recovered after the first week of VD and by day 11 post-VD the amplitude of tonic and busting EMG was 79 ± 10 µV and 85 ± 5 µV, respectively. The amplitude did not reach values of SH-VD.

The CMG presented double peaks during contraction (Fig. 6A). Immediately (day 0) and during the first 3 days after VD, the CMG showed a single peak during voiding (Fig. 6A).The values for CMG parameters were also significantly reduced (Fig. 6C). Voiding efficiency was significantly decreased in both 0–3, and 6–8 days after VD (*p* < 0.05, Fig. 6C).

### Experiment 4. Facilitated recovery of urinary continence by TENS of the DNC

**Figure 7:**
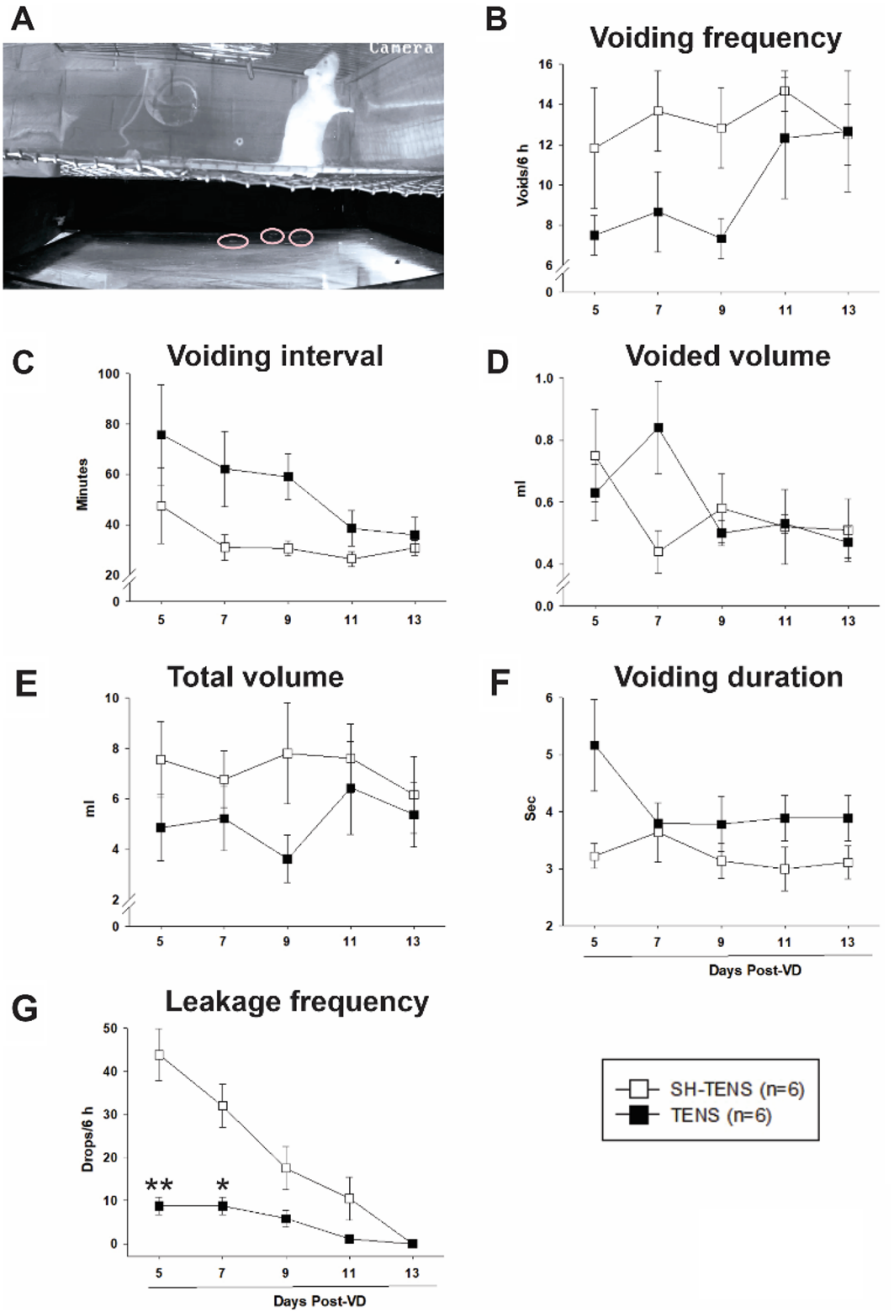
Urinary parameters of rats treated with transcutaneous electrical nerve stimulation (TENS) after vaginal distension (VD). (**A**) Photograph of a female rat standing on the hind-legs for vertical exploration with concomitant urine leakage (ovals indicate the drops expelled). Urinary parameters during the dark phase after VD or sham VD (SH-VD) during TENS or sham TENS (SH-TENS) treatments, including voiding frequency (**B**), voiding interval (**C**), voided volume (**D**), total voided volume (**E**), and voiding duration (**F**). (**G**) Urine leakage frequency as the number of drops in 6 h at different time points after VD with TENS or SH-TENS treatments. Values indicate mean ± standard error of the mean. *(*p* < 0.05), and ** (*p* < 0.01) indicate significant differences for TENS values vs. SH-TENS on the same time point (two way repeated measures ANOVA).

After VD both SH-TENS and TENS treated rats showed micturition behavior and also leaked urine during behaviors associated with effort, such as standing up on two feet (Fig. 7A). In comparison to SH-TENS, TENS significantly decreased the number of drops 5 and 7 days after VD (*p* < 0.001, Fig. 7G). Also, urine leakage after TENS disappeared earlier than in SH-TENS rats (Fig. 7G; compare day 11 vs. day 13). However, no significant differences were observed for the values of urinary parameters among SH-TENS and TENS (*p* > 0.05, Fig. 7B–F).

## Discussion

Parturition is a risk factor for the development of urinary incontinence in women^1,17^. Since invasive studies are not ethical in women, the pathophysiological mechanisms of urinary incontinence are not well stablished and treatments are limited. The present study unveiled the time-course of recovery of urinary continence and EUS neuromuscular function, as well as the mechanisms of EUS dysfunction after VD in a rat model. It also demonstrates that urinary continence recovery can be facilitated by TENS of the DNC, via a non-invasive method.

As in other studies^6,18^, we used urine leakage as indicative of behavioral signs of urinary incontinence. This method, combined with histological, histochemistry and electrophysiological studies demonstrated a relationship between behavioral signs of urinary incontinence and VD neuromuscular urethral tissue damage, with the maximum expression of behavioral and structural signs of damage 3–5 days after VD. Our results show that the abolition of the behavioral signs of incontinence was gradual and was further related to functional recovery of EUS neuromuscular circuitry. Recovery of urinary continence occurs by days 12–13 post-VD, and seems to be associated with gradual functional and histological recovery of the urethral components. However it is important to note that EUS was not fully recovered by day 11 post-VD, as indicated by mild EUS fibers fragmentation and decreased EUS EMG amplitude during bladder contraction. It is unknown whether under a stress challenge the urethral pressure of these rats would be able to maintain urine continence. In addition, urethral epithelia, which was fully recovered and partial recovered of the urethral musculature could underlie the recovery of urinary continence.

The current combination of methods allowed us to better understand the mechanisms of VD dysfunction, adding information about EUS nerve impairment and recovery, and a pre-clinical treatment for the facilitation of urinary continence after VD. Our behavioral, electrophysiological and histological results suggest that urinary incontinence can appear as a result of damage to several urethral structures, specifically: smooth and striated fibers, and the terminal nerve fibers of the MBPN. This is in concordance with a previous study showing significant stretching of this nerve immediately after VD, around 50% of the distal length located over the ventral wall of the vagina^6^.

The mechanisms for VD-induced EUS injury involve a direct compression of the urethra on the pubic bone^6^ and overdistension, followed by hypoxia, ischemia^19^ and inflammation^20^, which could lead to partial EUS muscle degeneration, mainly of the fibers attached to the vagina. As well as injury of nerve fibers and neuromuscular junctions^21^. All these factors can explain fragmentation of EUS fibers observed in histology and a significant reduction of the positively-stained AchE area immediately after VD and persistent until 5 days after VD. In concordance with degeneration of EUS fibers, inflammation, necrosis, and phagocytosis of hind limb striated muscle fibers by monocyte/macrophages has been demonstrated during the first week after VD injury in mice^22^ and rats^23^.

We found abolition of the EUS EMG activity during the clitoral-EUS reflex immediately after VD. The fact that the activity of the DNC, the afferent pathway of the clitoral-EUS reflex, was reduced but not abolished suggests neuropraxia and that some axons preserved their integrity and were still conducting action potentials. In addition, recovery of DNC activity occurred rapidly, 3 days after VD. This was very important for this study, since we applied electrical stimulation of the clitoris as therapy to facilitate urinary continence.

In contrast to the afferent pathway of the reflex, the efferent component was fully compromised, with signs of damage to EUS fibers and nerve degeneration. The magnitude of the EUS nerve stretching during VD (50%) is higher than the allowed nerve stretch for full recovery (15%)^24^, which suggest neurotmesis of terminal branches. The fact that the MBPN activity does not disappear during the clitoral-EUS reflex immediately after VD suggests that nerves proximal to the site of distension are not significantly injured.

The fact that VD rats did not leak urine by 12 days post-VD, indicates that the recovery of the urethral system was achieved during this time window. Potential processes may include urethral smooth and striated muscle regeneration, proliferation and differentiation of new striated fibers, as well as nerve fiber elongation and reinnervation. In support of these mechanisms, we observed the reappearance of the fibers attached to the vagina in AChE treated genitourinary tracts, indicating a regeneration process of the EUS two weeks after vaginal distension. It has been reported that regenerative processes of the striated muscle fibers start after the first week after injury. The mechanisms include macrophage activation, stimulation of myogenesis and fiber growth, proliferation of satellite cells, secretion of cytokines and growth factors by myocytes, such as IL-10, Arg-1 and IGF-1^25,26^.

It is important to note that although EUS neuromuscular circuit function gradually recovered, a portion of the EUS fibers remained injured 11–12 days after VD, indicating that the EUS remains in a regenerative process that continues longer. In fact, striated fiber regeneration can last for more than one month^21,22^, and some fibers may not be functional again.

EUS EMG activity showed recovery from ~ 27% at 6 days to 75% for bladder-EUS reflex and 90% for clitoral-EUS reflex at 11 days after VD, which correlated with improvement of urinary continence and voiding efficiency. A decrease in voiding efficiency immediately and 6–8 days after VD can be due to the loss of EUS muscular activity, as well as a potential decrease in bladder contractility. Thus, in addition to the loss of EUS bursting activity there is a concomitant VD-induced bladder impairment due to bladder overdistension. These could explain a decrease in bladder contraction amplitude and duration 3 days after VD. Since injury to the detrusor immediately after VD has been reported^6^.

Considering that two lifespan-weeks in rats correspond to approximately one year in women^27^, the urinary continence recovery of the VD rats after two weeks are in contrast with the fact that many women show a higher rate (> 50%) of urinary incontinence even at 1 year after delivery^28^. However, our model did not take into account the effects of the pregnancy on urinary continence mechanisms, as urinary incontinence during pregnancy is the greatest single predictor for stress urinary incontinence later in life^29^. Consequently, treatments to accelerate the recovery of urinary continence are necessary to prevent urinary incontinence later in life.

Our results showed that TENS of the DNC, a non-invasive neuromodulation method, facilitates urinary continence recovery, and hence accelerates the physiological processes of EUS neuromuscular regeneration. Although the precise mechanism is unknown, we suggest that TENS of the clitoral sheath activates DNC neurons, which in turn, transynaptically activate EUS motoneurons at the Onuf´s nucleus in the lumbosacral spinal cord^30^. Activation of EUS motoneurons can increase the expression of neurotrophic factors, as observed in studies after electrical stimulation of the PDN following VD^8,9^, and the genito-EUS reflex is also discharged by mechanical stimulation of the clitoral skin and vaginal tract^11,31^. Further studies are necessary to determine whether mechanical stimulation of the mentioned structures could facilitate nerve regeneration after VD.

In our studies three TENS episodes were enough to significantly decrease the signs of urinary incontinence in the VD rats. This finding suggests that this method has similar results to multiple stimulations of the PDN to improve urethral function and accelerate recovery from stress urinary incontinence after childbirth injury model in rats^10^. Multiple TENS of the DNC and electrical stimulations of the PDN, could serve as possible regenerative therapies for women with postpartum urinary incontinence and to alleviate this urinary pathology later in life.

Some limitations of this study are that the hormonal condition of the rats was unknown, and also the use of a quadruped animal model. However, it has been shown that the estrous cycle does not affect VD-induced urinary incontinence in mice^32^; and that reliable signs of urinary incontinence were observed in our awake quadruped animals. Another limitation is the short term recordings. Thus, although micturition function seems to be fully recovered, traumatic and ischemic injury of the urethra can induce loss of striated muscle fibers, which are replaced with fibrotic scar tissue^33^. Degeneration and fibrotic urethral processes can lead to the reappearance of postpartum urinary incontinence after years^34^, or months later in the case of the rats used in this study.

## Conclusions

We demonstrated that rat urinary continence function after VD requires 2 weeks to recover. VD traumatic-injury of the urethra neuromuscular circuitry recovered gradually during two week time window, however EUS fibers do not fully recover. Furthermore, TENS facilitated urinary continence recovery in rats after VD. Treatment to facilitate neuroregeneration should be applied within one-week after damage. Given that rats are the most used laboratory animal for urological studies, this information should be considered when designing experiments to assess treatments for neuro- and muscle regeneration after VD. Additional studies are necessary to assess if TENS could facilitate urinary continence recovery in women, and decrease urinary incontinence intensity after parturition.
